# Mesenchymal Stromal/Stem Cell Therapy Improves Salivary Flow Rate in Radiation-Induced Salivary Gland Hypofunction in Preclinical in vivo Models: A Systematic Review and Meta-Analysis

**DOI:** 10.1007/s12015-024-10700-y

**Published:** 2024-03-02

**Authors:** Amanda-Louise Fenger Carlander, Anders Kierkegaard Gundestrup, Per Marcus Jansson, Bjarke Follin, Cecilie Hoeeg, Birgitte Saima Kousholt, Rasmus Tolstrup Larsen, Kathrine Kronberg Jakobsen, Susie Rimborg, Anne Fischer-Nielsen, Christian Grønhøj, Christian von Buchwald, Charlotte Duch Lynggaard

**Affiliations:** 1 Department of Otolaryngology and Audiology, Head and Neck Surgery, Rigshospitalet, Copenhagen University Hospital, Copenhagen, Denmark; 2 Cardiology Stem Cell Centre, Rigshospitalet, Copenhagen University Hospital, Copenhagen, Denmark; 3 Department of Clinical Medicine, Aarhus University Group for Understanding Systematic Reviews and Meta analyses in Translational Preclinical Science, Aarhus University, Copenhagen, Denmark; 4 Department of Occupational Therapy and Physiotherapy, Rigshospitalet, Copenhagen University Hospital, Copenhagen, Denmark; 5 Section of Social Medicine, Department of Public Health, University of Copenhagen, Copenhagen, Denmark; 6 The Royal Danish Library, Copenhagen University Library, Copenhagen, Denmark; 7 Department of Immunology, Rigshospitalet, Copenhagen University Hospital, Copenhagen, Denmark; 8 Department of Otolaryngology, Head and Neck Surgery and Audiology, Rigshospitalet, Copenhagen University hospital, Copenhagen, Denmark

**Keywords:** Xerostomia, Radiotherapy, Cell Therapy, Mesenchymal stem Cells, Systematic Review

## Abstract

**Background:**

Mesenchymal stromal/stem cells (MSCs) have been suggested for salivary gland (SG) restoration following radio-induced salivary gland damage. This study aimed to determine the safety and effectiveness of MSC therapy on radio-induced SG damage and hypofunction in preclinical in vivo studies.

**Methods:**

PubMed and EMBASE were systematically searched for preclinical in vivo interventional studies evaluating efficacy and safety of MSC treatment following radio-induced salivary gland damage published before 10th of January 2022. The primary endpoint was salivary flow rate (SFR) evaluated in a meta-analysis. The study protocol was published and registered on PROSPERO (www.crd.ac.uk/prospero), registration number CRD42021227336.

**Results:**

A total of 16 preclinical in vivo studies were included for qualitative analysis (858 experimental animals) and 13 in the meta-analysis (404 experimental animals). MSCs originated from bone marrow (four studies), adipose tissue (10 studies) and salivary gland tissue (two studies) and were administered intravenously (three studies), intra-glandularly (11 studies) or subcutaneously (one study). No serious adverse events were reported. The overall effect on SFR was significantly increased with a standardized mean difference (SMD) of 6.99 (95% CI: 2.55–11.42). Studies reported improvements in acinar tissue, vascular areas and paracrine factors.

**Conclusion:**

In conclusion, this systematic review and meta-analysis showed a significant effect of MSC therapy for restoring SG functioning and regenerating SG tissue following radiotherapy in preclinical in vivo studies without serious adverse events. MSC therapy holds significant therapeutic potential in the treatment of radio-induced xerostomia, but comprehensive, randomized, clinical trials in humans are required to ascertain their efficacy in a clinical setting.

**Graphical Abstract:**

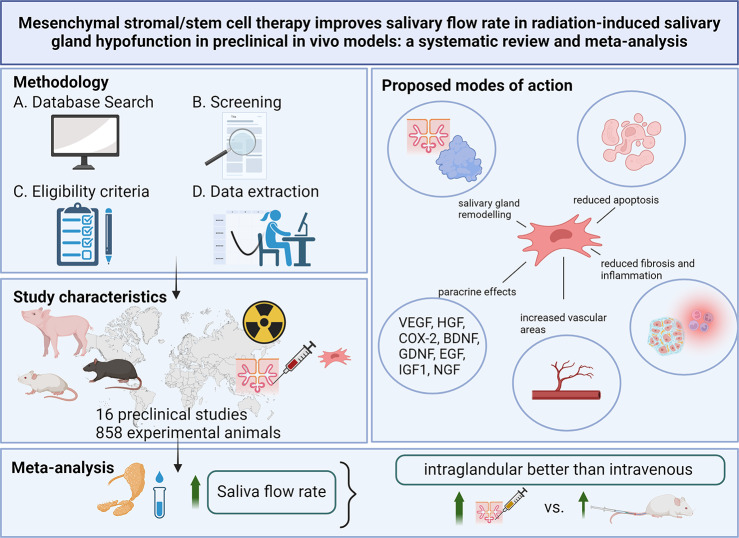

**Supplementary Information:**

The online version contains supplementary material available at 10.1007/s12015-024-10700-y.

## Introduction

Most patients with head and neck cancer (HNC) are treated with radiotherapy [1]. Salivary gland (SG) hypofunction and xerostomia, the subjective feeling of dry mouth, are common and long-term side effects following radiotherapy in the head and neck area [2]. Despite the emergence of intensity-modulated radiation-therapy (IMRT), that to some extent spare the surrounding tissue due to a more precise delivery to target tissue, the SG are often damaged [3, 4]. Radio-induced SG damage is dose-dependent and leads to gland degeneration and progressive decline in saliva production, followed by complications such as xerostomia, problems with speech and swallowing, oral infections and dental caries thus reducing quality of life. Currently, only symptomatic treatments are available, and there is a lack of regenerative and restorative therapeutic options [2, 5–7].

Mesenchymal stromal/stem cells (MSCs) are multipotent adult progenitor cells that in vitro can differentiate into mesodermal lineages with abilities for tissue regeneration and which can be isolated from numerous connective tissues, e.g. bone marrow (MSCs[M]) and adipose tissue (MSCs[AT]) [8–10]. Aside from being easily accessible, MSCs encompass various advantages such as proliferative and differentiating capacities; but also, immunomodulatory, and trophic properties such as anti-inflammatory, anti-fibrotic, anti-apoptotic, angiogenetic and immunosuppressive effects [11–13]. Thus, MSCs have a therapeutic and disease-modifying potential to repair and/or restore radio-induced SG damage. Recent preclinical in vivo studies have focused on mesenchymal stem cell (MSC) transplantation to repair radiation damaged SGs as a potentially curative treatment for SG hypofunction [14, 15]. Also, MSC therapy have shown to improve salivary flow rate (SFR) in humans [16–19].

Nevertheless, while MSC therapy shows potential as a treatment option for radio-induced SG damage, existing studies have been limited in size, characterized by high heterogeneity in relation to MSC origin, and only a few have been conducted in both preclinical in vivo models and humans. The use of MSCs therapy for radio-induced SG hypofunction alone has not yet been evaluated in a systematic review and meta-analysis. The aim of this study was therefore to review the safety and effectiveness of MSC therapy for restoring SG function after radiation-induced damage in preclinical in vivo studies. This is of great importance to optimize clinical trials and to assess the prospective implication in the curative treatment of SG hypofunction caused by radiotherapy.

## Method

The study adheres to the Preferred Reporting Items for Systematic Reviews and Meta-Analyses Protocols (PRISMA-P) statement and the study protocol was published and registered on PROSPERO (www.crd.ac.uk/prospero), registration number CRD42021227336 [20].

We considered preclinical in vivo models that assessed MSC therapy following experimentally induced radiation injury of major SGs. Inclusion criteria were: (1) preclinical in vivo intervention studies of both sexes and all ages (2) exposure of SGs to ionizing radiation (3) MSC therapy of all administration routes. There were no restrictions regarding induction of radiation damage, but MSCs administration should be after induction of radiation injury. MSC secretome, exosomes, and treatment with parts of MSCs were also included. In vitro models, treatments other than MSCs, SG damage other than ionizing radiation and non-relevant outcome were excluded. Human studies were not included in the formal analysis but described and included the discussion. The primary outcome was efficacy measured by SFR and secondary outcomes was SG morphology, SG histology, changes in saliva composition, circulating immune cells, SG paracrine effects, mode of action and safety in terms of objective adverse events as previously described [20].

### Systematic Search

In January 2022 two authors (ALFC, CH) systematically searched PubMed and Embase for preclinical in vivo interventional studies assessing the efficacy and safety of MSC therapy for radiation-induced SG hypofunction. The search was performed using Medical Subject Headings (MeSH), Emtrees and text words relating to MSCs, SG hypofunction, SG damage, SG dysfunction, radiation-induced SG damage or xerostomia. The specific search string for PubMed and Embase was previously described [20]. The search of databases reference lists was evaluated for additional relevant studies.

### Data Extraction

Two authors (ALFC, CH) independently screened all articles for eligibility and disagreement was solved by consensus or by discussion with a third reviewer (BM or CDL). The following information was extracted from each study: (1) article information (author, publication year), (2) details on preclinical in vivo model (species, sex, sample size, age), (3) study design (controlled, uncontrolled, randomized and/or blinded), (4) irradiation details (dose, Gy, quality assurance and days from irradiation to MSC therapy), 6) MSC therapy (type, concentration, administration route, follow-up time), 7) statistical analysis, 8) outcomes (functional and molecular outcomes).

### Quality Assessment and Risk of Bias

We assessed the quality of reporting in the included studies according to the latest Animal Research: reporting of in vivo Experiments (ARRIVE) guidelines [21]. One point was given for evidence of each quality criterion. The methodological quality was assessed using the SYRCLE (Systematic review Center for Laboratory Animal Experimentation) risk of bias tool in domains related to selection bias, performance bias, detection bias, attrition bias and reporting bias [22].

### Data Analysis

A descriptive summary of all outcomes was performed. The efficacy of MSC therapy was evaluated by a random effect meta-analysis adjusted to Hedge’s g on SFR. If there were multiple time points, only the last one was included in the meta-analysis. A standardized mean difference (SMD) with 95% confidence intervals (CIs) was used to evaluate the effect on SFR. The SMD was calculated by dividing the difference in mean outcome between the groups with the standard deviation of outcome among participants as per recommendation by the Cochrane Handbook [23]. Heterogeneity of the study results was investigated using the Cochrane Q test and quantified with I² values. Subgroup analyses were performed for species, strain, sex, administration route, age in weeks, radiation duration, frequency of treatment, radiation dose and time between radiation to MSC treatment. Sub analysis on frequency of radiation was not performed since radiation was administered as a single treatment in all studies.

## Results

**Fig. 1:**
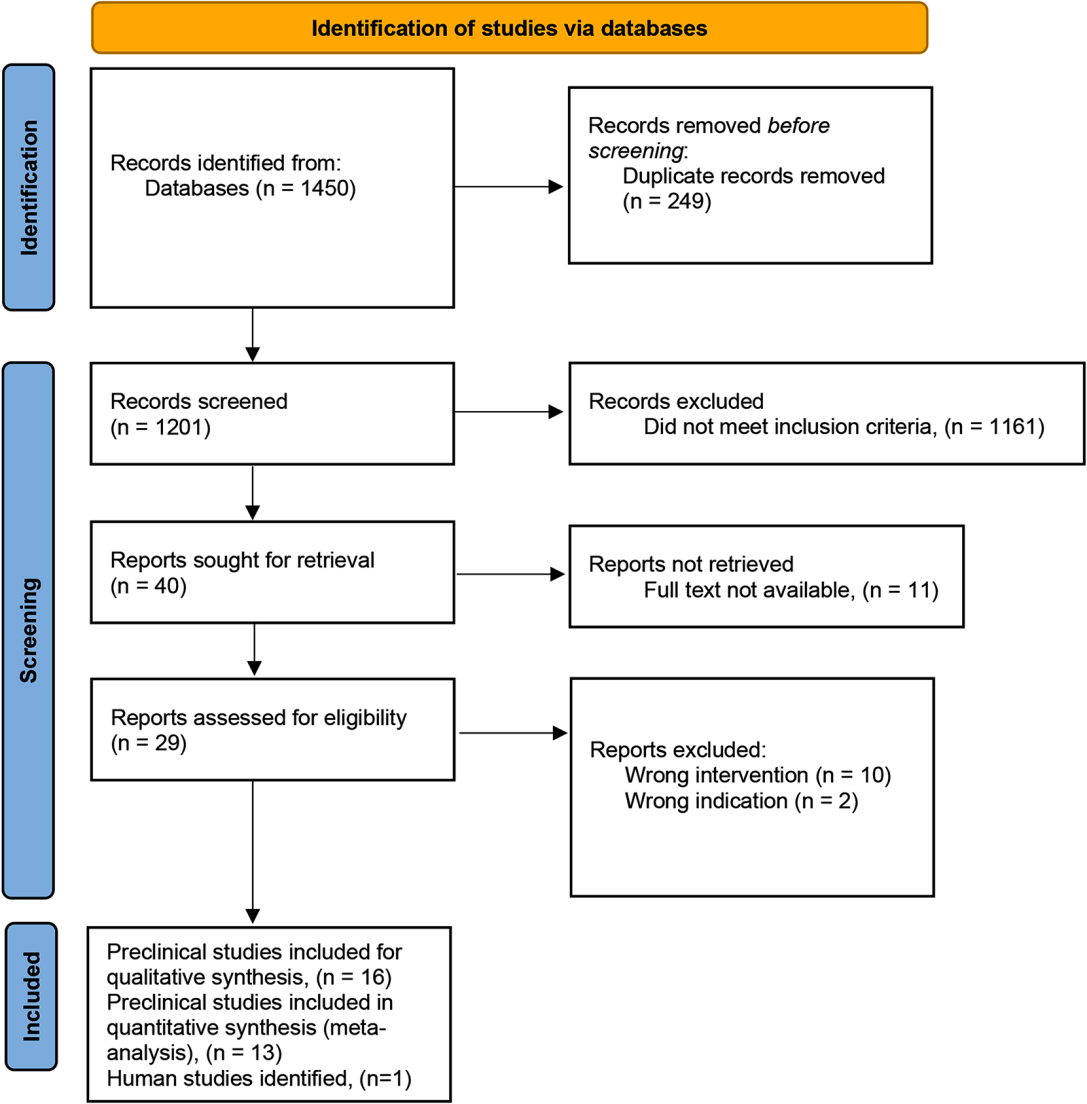
PRISMA 2020 Statement flow chart of the screening process for the selection of eligible studies

Sixteen preclinical studies published between 2011 and 2019 met the inclusion criteria, see Fig. 1 [24–39]. One human study [19] (*n* = 30, intervention group *n* = 15) were identified. All preclinical in vivo studies were included in the quantitative (858 experimental animals) and 13 (404 experimental animals) were also included in the qualitative analyses, see Fig. 1.

### Description of the Preclinical in vivo Models

All studies investigated the safety and efficacy of MSC-based therapy for xerostomia and SG hypofunction following radiation in the head and neck area. A total of 858 animals were included of which 341 received intervention with MSC therapy. Ten studies included mice, two studies rats and one study miniature pigs. The irradiation dose varied from 10 to 25 Gy, and all were administered as a single dose. See Table 1.

**Table 1:** Study characteristics

Author (Year)	Animal: species, strain and age, gender	Study design	Groups	Irradiation	Days from radiation to MSC treatment	MSC type and concentration	Administration route	Statistical analysis	Functional outcome	Molecular outcome
Lin, C et al. (2011)	Mice, NOD.SCID-*Prck*^*SCID*^, four weeks old	Prospective, controlled trial	1. Control group, no IR, no treatment, *n* = 35. 2. IR, no treatment, *n* = 35. 3. IR, treated with MSC(M), *n* = 35. 4. IR, treated with acinar-like cells, *n* = 35	Single dose, 15 Gy, from ^60^Co-source. No QA on radiation delivery was performed.	11 days	10^6^ MSC(M) and acinar-like cells, co-cultured from MSC(M).	Intraglandular injection (submandibular gland)	One-way ANOVA and marginal linear regression analysis. Significance level was *P* < .05.	Saliva production in group 3 and 4 significantly increased. Body weight and gland weight significantly higher in groups 3 and 4. Significantly faster rate of recovery in gland weight in group 4, compared to group 3.	No molecular data. However, a significantly higher expression of ${\upalpha }$-amylase in acinar cells and co-cultured MSC(M) compared to MSC(M) which was not been co-cultured
Kojima, T. et al. (2011)	Mice, C57BL/6, nine weeks old	Prospective, controlled trial	1. Control group, no IR, no treatment, *n* = 24. 2. IR, treated with ADSCs in PBS, *n* = 31. 3. IR, treated with PBS only, *n* = 31.	Single dose, 10 Gy gamma irradiation from ^137^C source. No QA on radiation delivery was performed.	70 days (10 weeks)	ADSCs 0.1 mL solution of PBS containing 500.000 cells.	Intraglandular injection (submandibular glands)	One-way and two-way ANOVA followed by Fishers PLSD or students *t*-test. Significance level was *P* < .01 or *P* < .05.	Significant increase in SFR in ADSC group compared to sham. 75% function compared to normal group.	Group 2 had more acinar cells and less inflammatory infiltration compared to group 3. Group 2 had significantly more CD31-positivity, and showed a significant increase in levels of HGF, VEGF and several proteins related to angiogenesis.
Jeong, J. et al. (2013)	Rats, Wistar rats, six weeks old, male	Prospective, controlled trial	1. Control group, no IR, no treatment. 2. IR, treated with PBS 3. IR, treated with hSGSCs	Single dose, 25 Gy, dose rate of 2 Gy min^− 1^. No QA on radiation delivery was performed.	1 day	5 × 10^5^ hSGSCs	Intraglandular injection	One-way ANOVA. Significance level of < 0.05 was used.	At 60 days, significant increase in SFR and body weight in group 3 compared to group 4.	Group 3 had compact acinar and ductal structure like undamaged rat tissue, whereas group 2 had disrupted acinar structure and numerous vacuoles. Apoptotic cells were observed only in group 2 and not in group 3.
Lim, J. et al. (2013)	Mice, C57BL/6, eight to nine weeks old	Prospective, randomized, controlled trial.	1. Control group, no IR, no treatment, *n* = 8. 2. IR, treated with PBS, *n* = 8. 3. IR, treated with BM-cMSCs, *n* = 8.	Single dose, 15 Gy. No QA on radiation delivery was performed.	1 day	1 × 10^5^ cMSC(M) in 15 µL of PBS.	Intraglandular injection (submandibular glands)	Mann-Whitney test for differences between the two groups. Kruskal-Wallis followed by post hoc Dunns for three groups. Significance level was *P* < .05.	Significant increase in SFR in group 3 compared to group 2. Body weight, gland weight and salivary lag time showed no significant differences.	Significant reduction of apoptosis and increase in microvessel density in group 3 compared to group 2. No significant difference in proliferation activity. Significantly higher mucopolysaccharide and tissue amylase upon histological examination.
Lim, J. et al. (2013)	Mice, CH3, eight to nine weeks old	Prospective, randomized, controlled trial.	1. Control group, no IR, no treatment, *n* = 20. 2. IR, no treatment, *n* = 20. 3. IR, treated with hAdMSCs, *n* = 20.	Single dose, 15 Gy No QA on radiation delivery was performed.	0 days (once weekly for 3 consecutive weeks)	1 × 10^6^ MSC(AT)h.	Intravenously (tail vein)	Mann-Whitney test for differences between two groups. Kruskal-Wallis followed by post hoc Dunns for three groups. Significance level was *P* < .05.	At 12 weeks significant increase in SFR in group 3 compared to group 2. Salivary lag time was significantly longer in group 2 compared to group 1.	A significantly higher fraction of mucin and amylase and reduction in apoptosis in group 3 compared to group 2. No significant differences in proliferative activity.
Xiong, X. et al. (2014)	Rats, Spraque-Dawley, 12 weeks old	Prospective, randomized, controlled trial.	1. Control group, no IR, no treatment, *n* = 30. 2. IR, treated with PBS, *n* = 30. 3. IR, treated with hADSCs, *n* = 30.	Single dose, 18 Gy, dose rate of 300 cGy min^− 1^ No QA on radiation delivery was performed.	0 days	1 × 10^6^ MSC(AT)h in 0.1 mL PBS solution.	Intraglandular injection (submandibular glands)	One-way ANOVA and Student-Newman-Keuls analysis. Significance level was *P* < .05.	At 24 weeks, significant increase in SFR in group 3 compared to group 2. Group 3 recovered to 71% SFR compared to group 1.	Group 3 had a significantly larger number of acinar cells and PAS-positive acini, significantly higher area of blood vessels and significantly fewer apoptotic cells compared to group 2. Significant increase in mRNA levels of VEGF, HGF, COX-2 and MMP-2 in group 3 compared to group 2.
Chen, Y. and Niu, Z. et al. (2014)	Minipigs, eight months old, female	Prospective, randomized, controlled trial.	1. IR, treated with ADSCs + PRF, *n* = 5 2. IR, treated with ADSCs, *n* = 5 3. IR, treated with PRF, *n* = 5 4. IR, treated with PBS, *n* = 5	Single dose, 20 Gy, dose rate of 3 Gy min^− 1^. Only right parotid gland was radiated. No QA on radiation delivery was performed.	28 days (four weeks)	4 × 10^6^ cells ADSCs (autologous),	40 subcutaneous injections points (0.2 mL/point), with 2 cm interval between neighbouring points.	One-way ANOVA and Kruskal-Wallis H test. Significance level was *P* < .05.		Group 1 showed significantly more fat cells, less fibrosis and inflammation compared to group 4. Group 2 and 3 were significantly better compared to group 4. Group 1–3 showed significantly less apoptotic activity compared to group 4 and group 1 showed the least. Group 1–3 showed significantly more neovascular capillary area, compared to group 4 and group 1 showed the most.
An, H. et al. (2015)	Mice, CH3, eight to nine weeks old	Prospective, controlled trial	1. Control group, no IR, no treatment, *n* = 35. 2. IR, treated with PBS, *n* = 35. 3. IR, treated with hADMSC SEC, *n* = 35	Single dose, 15 Gy. No QA on radiation delivery was performed.	0 days (once daily for seven consecutive days)	500 µL MSC(AT)h. Sectretome stemmed from 1 × 10^5^ cell.	Intravenously (tail vein)	Mann-Whitney test, one-way ANOVA followed by Tukey’s post hoc test, two-way ANOVA followed by Bonferroni post hoc test and linear regression. Significance level was *P* < .05.	At 16 weeks, significant increase in SFR in group 3 compared to group 2. Salivary lag time was significantly longer in group 3 compared to the other groups. Body and gland weight was significantly higher in group 3 compared to group 2.	Saliva amylase activity was significantly lower in group 2 compared to group 1, while group 3 was not. EGF content in saliva was significantly higher in group 3 compared to group 2. PAS stain for mucin showed significantly larger area of mucin in group 3 compared to group 2.
Li, Z. et al. (2015)	Mice, C57BL/6, eight weeks old	Prospective, randomized, controlled trial	1. IR, treated with ADSCs, *n* = 10 2. IR, treated with PBS, *n* = 10 3. Control, no IR, no treatment, *n* = 10	Single dose, 18 Gy No QA on radiation delivery was performed.	0 days (twice a week for six weeks)	1 × 10^6^ cells ADSCs.	Intravenously (tail vein)	ANOVA followed by Tukey’s honestly significant difference test. Significance level was *P* < .05.	At eight weeks significant increase in SFR in group 1 compared to group 2. Gland weight was significantly higher in group 1 compared to group 2.	PAS staining of glands showed significant increase in production of mucopolysaccharide, C31 staining showed significantly higher density of microvessels, and significantly higher levels of amylase in group 1 compared to group 2. Significantly higher proliferation activity and reduction of apoptosis activity in group 1 compared to group 2.
Wang, Z. et al. (2016)	Mice, C3H, 8–12 weeks old, female	Prospective, randomized, controlled trial	1. Control group, no IR, no treatment, *n* = 10 2. IR, treated with PBS, *n* = 10 3. IR, treated with ADSCs, *n* = 10 4. IR, treated with PR, *n* = 10 5. IR, treated with ADSCs + PRF, *n* = 10	Single dose, 18 Gy No QA on radiation delivery was performed.	84 days (12 weeks)	2 × 10^5^ ADSCs in 100 µL solution of PBS/PRF.	Intraglandular injection (submandibular glands). Weekly injections for 3 consecutive weeks.	Mann-Whitney test and one-way ANOVA followed by Tukey’s post hoc test. Significance level was *P* < .05.	At 12 weeks. Significant increase in SFR, body weight and gland weight in group 3, 4 and 5 compared to group 2.	Group 5 showed significantly more acinar cells, more amylase area compared to groups 2,3 and 4. Group 5 showed significantly more microvessels, compared to group 2 and 4, but not group 3. Group 5 showed less apoptotic activity and more proliferation activity compared to group 2.
Wang, Z. et al. (2017)	Miniature pigs, eight months old, female	Prospective, randomized, controlled trial	1. Control group, no IR, no treatment, *n* = 5 2. IR, treated with ADSCs, *n* = 5 3. IR, treated with PBS, *n* = 5	Single dose, 20 Gy, dose rate was 3.2 gy min^− 1^. Only right parotid gland was radiated. No QA on radiation delivery was performed.	0 days (un-clear?)	4 × 10^6^ ADSCs	Intraglandular injection at 40 different injection points, 0.2 mL per injection. Repeated twice per week for 6 weeks.	ANOVA followed by Tukey’s honestly significant difference test. Significance level was *P* < .05.	At 3 months significant increase in SFR and gland weight in group 2 compared to group 3. There was no significant difference in gland weight between group 1 and 2.	Amylase levels was significantly higher in group 2 compared to group 3. The number of PAS-positive acinar cells was significantly higher in group 2 compared to group 3. Group 2 showed less fibrosis and more vascularization than group 3.
Choi, J. et al. (2018)	Mice, C3H, eight to nine weeks old.	Prospective, randomized, controlled trial	1. Control group, no IR, no treatment, *n* = 28 2. IR, treated with PBS, *n* = 28 3. IR, treated with porcine SIS, *n* = 28 4. IR, treated with AdMSC, *n* = 28 5. IR, treated with AdMSC + SIS, *n* = 28	Single dose, 15 Gy No QA on radiation delivery was performed.	0 days	1 × 10^5^ cells MSC(AT) in 20 µL solution of respective carrier.	Intraglandular injection (submandibular glands)	T-test, one-way ANOVA followed by Tukey’s post hoc test. Significance level was *P* < .05.	At 16 weeks, significant increase in SFR in group 5 compared to group 2. Group 4 and 5 showed significantly lower lag-time compared to group 2.	Group 4 and 5 showed significantly higher levels of EGF and amylase compared group 2. Group 4 and 5 showed significantly less fibrosis than group 2 and 3. Only group 5 showed increased mucin production, compared to group 2 and 3. Group 4 and 5 showed significant anti-apoptotic effects and increased ROS scavenging effects compared to group 2.
Shin, H. et al. (2018)	Mice, C3H, six weeks old, female	Prospective, randomized, controlled trial	1. Control group, no IR, no treatment, *n* = 6 2. IR, treated with PBS, *n* = 6 3. IR, treated with SGSC^2D^, *n* = 6 4. IR, treated with SGSC^3D^, *n* = 6	Single dose, 15 Gy No QA on radiation delivery was performed.	28 days (four weeks)	2 × 10^5^ SGSC^3D^ SGSC^2D^ in 10 µL solution of PBS.	Intraglandular injection (submandibular glands)	Mann-Whitney test, one-way ANOVA followed by Tukey’s post hoc test, two-way ANOVA followed by Bonferroni post hoc test, as well as linear regression. Significance level was *P* < .05.	At 16 weeks, significant increase in SFR, body and gland weight and lower lag time in group 3 and 4 compared to group 2. Significant increase in SFR, body and gland weight and lower lag time in group 4 compared to group 3	Group 3 and 4 showed significantly higher amylase activity and higher levels of EGF compared to group 2. Group 4 showed significantly higher amylase activity and levels of EGF compared to group 3. Group 3 and 4 showed significantly higher expression of genes related to salivary gland function and growth factors compared to group 2. Group 4 showed significantly higher expression compared to group 3, however by a smaller relative margin.
Shin, H. et al. (2018)	Mice, C3H, six weeks old.	Prospective, randomized, controlled trial	1. Control group, no IR, no treatment, *n* = 4 2. IR, treated with PBS, *n* = 4 3. IR, treated with hADSC^NMX^, *n* = 4 4. IR, treated with hADSC^HPX^, *n* = 4	Single dose, 15 Gy. No QA on radiation delivery was performed.	28 days (four weeks)	2 × 10^5^ MSC(AT)h^HPX^ and MSC(AT)h^NMX^ in 10 µL solution of PBS.	Intraglandular injection (submandibular glands)	Mann-Whitney test, one-way ANOVA followed by Tukey’s post hoc test, two-way ANOVA followed by Bonferroni post hoc test. Significance level was *P* < .05.	At 12 weeks significant increase in SFR, **amylase activity**, body and gland weight in group 3 and 4 compared to group 2, and significantly higher in group 4 compared to group 3. Lag time was significantly shorter in group 3 & 4 compared to group 2.	Significantly more fluorescently labelled MSC(AT)h^HPX^ cells compared to MSC(AT)h ^NMX^ cells in random fields. The density of PAS-positive acinar cells was significantly higher in group 3 and 4 compared to group 2, and significantly higher in group 4 compared to group 3. Significantly less fibrosis in group 3 and 4 compared to group 2. EGF levels were higher in group 3 and 4 compared to group 2.
Elsaadany, B. et al. (2019)	Rats, albino, three to four months old, male	Prospective, randomized, controlled trial	1. Control group, no IR, no treatment, *n* = 6 2. IR, treated with PBS, *n* = 6 3. IR, treated with MSC(M), *n* = 6	Single dose, 13 Gy. No QA on radiation delivery was performed.	0 days	1 × 10^5^ MSC(M) in 0.2 mL PBS solution.	Intraglandular injection.	ANOVA followed by Tukey’s post hoc test. Significance level was *P* < .05.	No functional outcome parameter.	The acini diameter was significantly larger in group 3 compared to group 2. Group 3 showed significantly less fibrosis compared to group 2. Group 2 showed necrosis of the ductal lining epithelium, which was less commonly observed in group 3, however no significantly different data was reported.
Mulyani, S. et al. (2019)	Rats, Wistar, three to four months old, male	Prospective, randomized, controlled trial	1. Control group, no IR, no treatment, *n* = 10 2. IR, no treatment, *n* = 10 3. IR, treated with MSC(M)^NMX^, *n* = 10 4. IR, treated with MSC(M)^HPX^, *n* = 10	Single dose, 15 Gy. No QA on radiation delivery was performed.	1 day	MSC(M)^HPX^ and MSC(M)^NMX^. Dose not specified.	Not specified	Normality test and MANOVA test. Significance level was *P* < .05.	At 7 days, group 4 showed a significantly higher expression of amylase compared to group 3. Group 3 and 4 showed significantly higher levels compared to group 2.	The expression of SDF1-CXCR4 and Bcl-2 was significantly higher in group 4 compared to group 3.

Abbreviations: ADSC, adipose-derived stromal cells; ANOVA, analysis of variance; Bcl-2, B-cell lymphoma 2; COX-2, cyclooxygenase-2; EGF, epidermal growth factor; HGF, hepatocyte growth factor; hADSC, human adipose-derived stromal cells; hSGCs, human salivary gland stem cells; IR, irradiation; MANOVA, multivariate analysis of variance; MMP-2, matrix-metalloproteinase-2; MSC(AT)h, human adipose-derived mesenchymal stem cells; MSC(AT)h SEC, human adipose-derived mesenchymal cell secretome; MSC(AT)h^HPX^, human adipose-derived mesenchymal stem cells incubated in a hypoxic environment; MSC(AT)h^NMX^, human adipose-derived mesenchymal stem cells incubated in a normoxic environment; MSC(M), bone marrow-derived clonal mesenchymal stem cells; MSC(M)^HPX^, bone marrow-derived mesenchymal stem cells incubated in a hypoxic environment; MSC(M)^NMX^, bone marrow-derived mesenchymal stem cells incubated in a normoxic environment; PBS, phosphate-buffered saline (PBS) solution; PAS, Periodic acid Schiff; PRF, platelet-rich fibrin; SGSC2D, human parotid gland 2D monolayer cultured human salivary gland stem cells; SGSC3D, 3D spheroid microwell cultured human salivary gland stem cells; SDF1-CXCR4, stromal cell-derived factor 1-C-X-C chemokine receptor type 4; SFR, salivary flow rate; SIS, small intestine submucosa; VEGF, vascular endothelial growth factor; QA, quality assurance

MSCs were originating from bone marrow (4 studies) [24, 27, 36, 37], adipose tissue (10 studies) [25, 28–33, 35, 38, 39], and salivary gland tissue (2 studies) [26, 34] and were administered intravenously (3 studies) [28, 31, 39], intra-glandularly (11 studies) [24–27, 29, 32–36, 38] or subcutaneously (1 study) [30]. One study did not specify administration route [37]. One study used secretomes originating from adipose tissue [39]. The mean follow-up time was 12 weeks after MSC treatment, ranging from 7 days to 24 weeks. See Table 1.

### Safety of MSC

All included preclinical in vivo studies described the MSC-based cell therapy as safe with no reported serious adverse events [24–39].

### The Effect of MSCs on Efficacy, Salivary Flow Rate

**Fig. 2:**
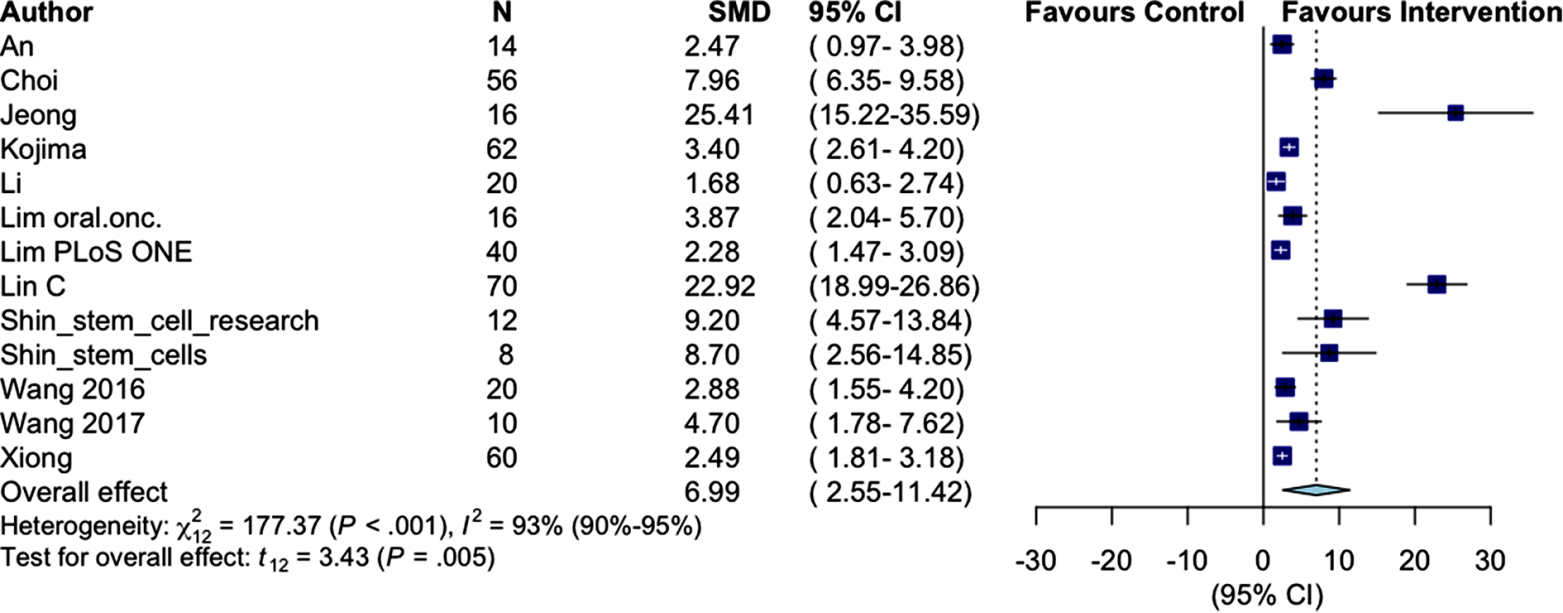
Random-effects meta-analysis of the overall effect on SFR following MSC therapy. SFR: salivary flow rate; SMD: standardized mean difference; MSC: mesenchymal stem cell

13 studies included SFR as primary functional outcome [24–29, 31–35, 38, 39]. Eleven studies found a significant increase in SFR after intervention with MSCs compared to other groups (placebo/sham or IR only) [24–29, 32–35, 38], with an overall effect of SMD 6.99 (95% CI: 2.55–11.42), ranging from 1.68 (95% CI: 0.63–2.74) [31] to 25.41(95% CI: 15.22–35.59) [33]. Heterogeneity was X^2^ = 177.37 (*p* < .001) and I^2^ = 93% (95% CI [90–95%]). See Fig. 2.

Subgroup analyses investigating the effect of species, strain, sex, administration route, age in weeks, radiation duration, frequency of treatment, radiation dose and time between radiation to MSC treatment revealed significant differences regarding strain (*p* < .001) and administration route (*p* = .01). The most prominent differences were observed in the strains NOD.SCID-PrckSCID (SMD 22.92, 95% CI: 18.99–26.86) and Wistar (SMD 25.41, 95% CI: 15.22–35.59) compared to C57BL/6 (SMD 2.90, 95% CI: 0.08–5.52), CH3 (SMD 4.99, 95% CI: 1.58–8.39) and Spraque-Dawley (SMD 2.49, 05% CI: 1.81–3.18), while the effect following intraglandular injection was greater than following intravenously injection (SMD 8.59, 95% CI: 2.94–14.24 versus 2.12, 95% CI: 1.20–3.05, respectively). There was no significant effect of species, sex, age in weeks, radiation dose, frequency of treatment or time from radiation to first treatment. Effect of duration of radiation was not possible to assess due to insufficient data. See **Supplementary Results**.

### The Effect of MSCs on Salivary Gland Regeneration and Apoptosis

Seven studies reported improvements in acinar tissue [25–27, 29, 31, 32, 35] with more acinar cells [25, 29, 32, 35] and more compact acinar structure [26]. Also, several studies reported less inflammation and fibrosis [25, 28, 30, 33, 35, 36] and increased amylase [27, 28, 31–34, 37–39]. Li et al. and Wang et al. reported intact cellular ultra-microstructure with healthy cell membrane and almost undamaged cytoplasmic organelles [31, 38]. They also found significantly higher proliferative activity, but this was not found by others [27, 28]. Shin et al. found greater expression of SG epithelial cell markers (KRT7 and KRT18) and upregulated structure-related genes (SMR3A, AMY2A5, PRB1, AMY1, CLDN22, PRPMP, AMY1A and AQP5) [35]. Similarly, Choi et al. found higher expressions of AQP5, alfa-SMA and CD31 [33]. Mulyani et al. found increased expression of SDF1-CXCR4 and Bcl-2 genes [37]. Nine studies reported reduction in apoptotic cells [26–33, 38], though Wang et al. did not specify the results [32].

Lim et al. observed anti-alfa-amylase signaling in transplanted MSC(M) suggesting a transdifferentiation into SG epithelial cells [27], but this was not found by Kojima et al. [25].

### The Effect of MSCs on Vascular Areas

Several studies reported an increase in vascular areas [25, 27, 29–31]. Furthermore, Kojima et al. localized MSCs in vessel endothelial cells post transplantation five- and ten-weeks post transplantation [25]. Wang et al. also found improvements in vascular areas, but only for the intervention group receiving both MSCs and platelet-rich-fibrin (PRF) [38].

### The Paracrine Effects of MSCs

Five studies reported on the paracrine effect of MSC treatment [25, 29, 33–35]: Xiong et el. found increased mRNA levels VEGF, HGF and COX-2 [29]; Shin et al. found higher expression of the paracrine factors BDNF, GDNF, EGF, IGF1 and NGF [34]; Shin et al. found greater expression of the growth factor FGF10 [35]; Kojima et al., found increased expressions of HGF, VEGF, COX-2 and MMP-2 [25] and Choi et al., found increased levels of EGF [33].

### Homing of Systemically Transplanted MSCs

Both Li et al. and Lim et al. found that systematically transplanted MSCs could be identified in the salivary glands post transplantation [28, 31]. An et al. also administered the therapy intravenously but did not report on homing to the SG post transplantation [39].

### Platelet-Rich Fibrin in Addition to MSCs

Two studies also investigated MSC + PRF [30, 38]. Chen et al. found the MSC + PRF group had significantly improvement on soft tissue defects [30], while Wang et al. found that MSC + PRF had increased levels of acinar cells, amylase, microvessels and proliferative activity and reduced levels of apoptotic cells [38].

### The Effect of MSCs on Efficacy and Safety in Human Studies

One human study investigated the intervention of intraglandular autologous MSCs(AT) in a randomized, placebo-controlled phase I/II study (*n* = 30) [19]. The study found MSC treatment to be safe and reported a significant increased UWS (50% after four months, *p* = .003) including improvements in patient-reported outcomes in the group receiving MSCs. Also, a significant increase in serous gland tissue, improvements in saliva composition, and a decrease in connective were observed. See **Supplementary Results**.

### Risk of Bias and Quality Assessment

**Fig. 3:**
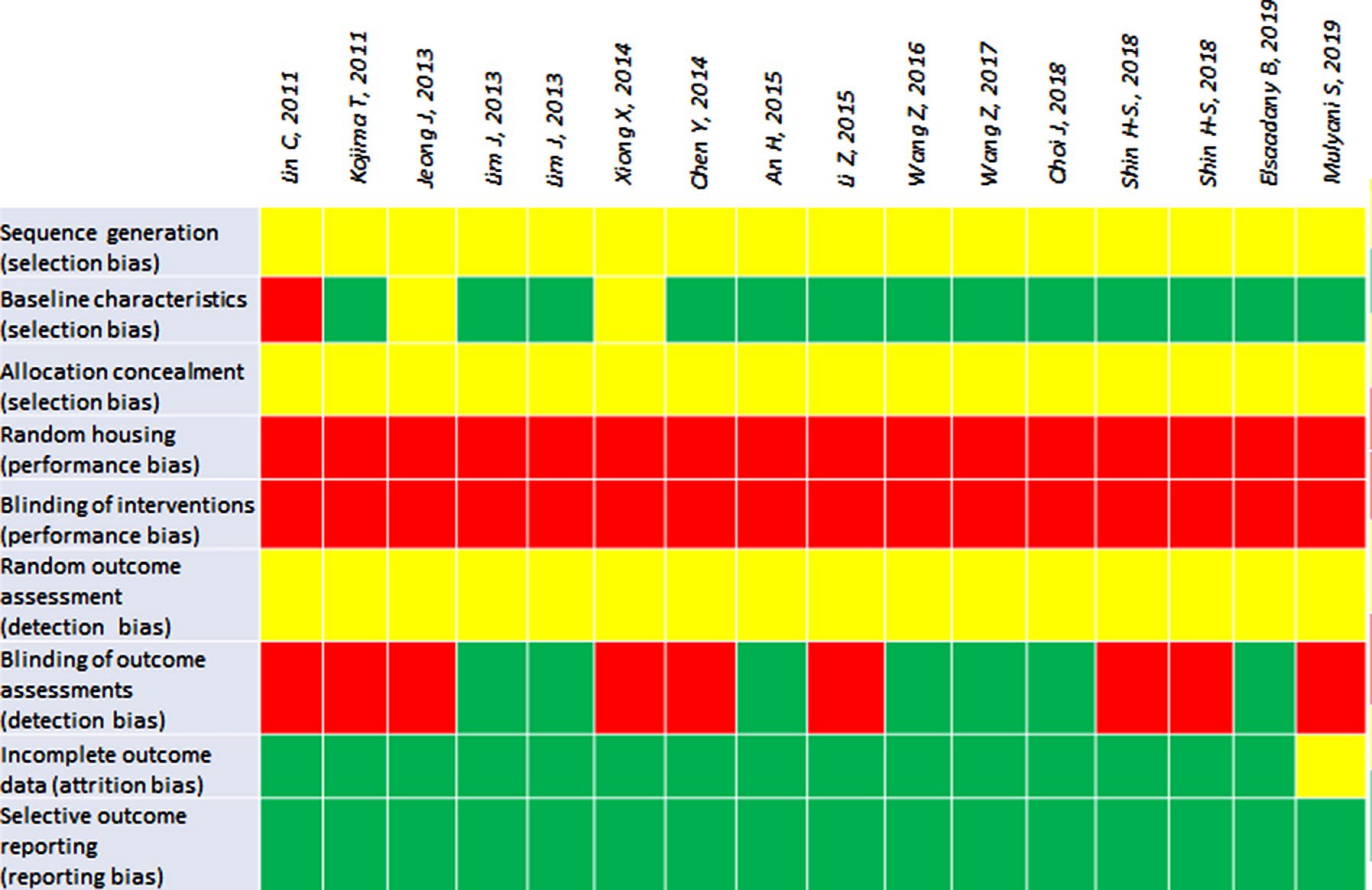
SYRCLEs Risk of bias assessment tool. *Green* = low risk of bias, *Yellow* = unclear risk of bias, *Red* = high risk of bias

The preclinical in vivo studies were assessed using SYRCLEs risk of bias assessment tool with nine questions to determine potential biases [22]. All studies involved a risk of bias, especially regarding selection bias as none of the studies reported how the randomization of intervention/control groups was performed [24–39]. Also, all studies revealed a high risk of performance bias, since none of the included studies used random housing or blinding of interventions/investigators [24–39]. All studies, except Mulyani et al., had a low risk of bias concerning attrition bias and reporting bias. Seven studies had a low risk of detection bias since they reported blinding of the outcome assessment [27, 28, 32, 33]: (1) histological examination [26, 35] (2) immunohistochemical evaluation [27, 31, 32, 37] (3) evaluation of apoptotic cells [26, 27, 32, 37, 38] (4) evaluation of cytoprotective effects (AQP5, CD31, alfa-SMA, c-Kit) [38]. Nine studies did not report blinding [24–26, 29–31, 34, 35, 37]. See Fig. 3.

The quality of the included studies was assessed using the ARRIVE guidelines [21]. Highest score given was 9 [27, 28, 32, 33, 36, 38] and lowest score given was 5 [26]. Most studies revealed high quality with ≥ 8 points [25, 27–29, 31–36, 38, 39], but 4 studies had a total score of ≤ 7 [24, 26, 30, 37]. All studies except Jeong et al. reported sufficiently regarding study design and sample size [24, 25, 27–38]. All studies reported sufficiently on outcome measures and results and all studies were randomized, though none reported on the randomization method used and none reported how they included/excluded animals [24–39]. One study failed to report sufficiently on statistical method [24], one on experimental animals [30] and two studies failed to report sufficiently on experimental procedures [26, 37]. See **Supplementary Results.**

## Discussion

In this systematic review and meta-analysis, MSC therapy demonstrated a significant effect on SG function following radiation-induced gland damage in preclinical in vivo studies. The treatment proved to be safe, with no reported adverse reactions.

We found that MSC therapy had a significant impact on the SG functioning with a significant increase in the SFR. However, there was a high heterogeneity among included studies with differences in MSC origin, species, strains, age, radiation dose, administration route of MSC therapy, frequency of treatment and time between radiation and first treatment. The impact on SFR was significantly associated with strain and administration route. The most pronounced effect on SFR was observed when MSCs were administered intraglandular compared to systemic transplantation. Whether the effect of MSC therapy varies by strain is difficult to conclude, as only one study included Wistar [26], and one study NOD.SCID-PrckSCID [24], which both exhibited the most significant effect.

All studies but one, by Lin et al., reported elements of SG remodeling properties such as: increased density of acinar cells, more compact acinar structure, increased levels of amylase, decreased inflammation and fibrosis. If MSC therapy induces a higher proliferative activity remains uncertain. While Shin et al. reported such an effect, it was not confirmed by other studies [26, 27]. As possible modes of action, upregulation in epithelial markers (KRT7 and KRT18) and structure-related genes (SMR3A, AMY2A5, PRB1, AMY1, CLDN22, PRPMP, AMY1A, AQP5, AQP5, alfa-SMA and CD31) was identified [33, 35], while another study by Mulyani et al. found upregulation in genes encoding for proteins involved in cell migration, survival and differentiation (SDF1-CXCR4 and Bcl-2) [37].

This study also indicates that increased blood vessel regeneration and paracrine functioning participate in the tissue repair and restoration of gland damage. Several studies reported an increase in vascular areas [24, 26, 28–30, 32]. A wide range of growth factors (VEGF, HGF, COX-2, BDNF, GDNF, EGF, IGF1, NGF, FGF10 and MMP-2) contributing to various aspects of regeneration including angiogenesis, neural regeneration and cellular proliferation were identified – ultimately supporting the repair of damaged glandular tissues.

The severity of radio-induced salivary gland damage is influenced by several factors, with radiation mean dose being a critical one [40]. The delivery of radiotherapy was greatly standardized and administered as a single dose in all the included studies, which does not resemble the clinical radiotherapy regimens for head and neck cancer which are patient-specific and often fractionated across several weeks. This is important to keep in mind, when translating the findings from this systematic review to a clinical setting. Prolonged exposure to radiation leads to cumulative damage, but unfortunately, we could not further investigate the relation between radiation duration and effect of MSC therapy due to insufficient data. The time from radiotherapy to administration of MSC might also be important, since especially the decreased levels of apoptotic cells found in several studies [26–28, 38], indicates that MSC therapy could be protective in the acute phase of radiotherapy. The timing of MSC administration varied across the studies from 0 days [28, 29, 31–33, 36, 39] to 12 weeks [25] post radiotherapy, but we observed no impact of the time from radiation to the initial MSC treatment.

Two studies that investigated the effect of intravenously administered MSC therapy also reported on homing to the SGs post transplantation, both identifying MSCs in the SGs [28, 31]. However, large-scale studies are required to further investigate the migration and homing following systemic transplantation.

PRF is a platelet-rich regenerative therapy containing a variety of growth factors and is known to promote cell proliferation [41]. Two studies investigated if additional PRF treatment improved SG function [30, 38]. Chen et al. found the MSC + PRF group had significantly improvement on soft tissue defects, but the groups were small, *n* = 5 [30]. Wang et al. found that interventions with MSC, PRF or MSC + PRF improved SFR, gland and body weight, but MSC + PRF performed better regarding the regenerative outcomes [38].

In addition, the preclinical in vivo studies included in the meta-analysis, we identified one human, phase I/II, randomized, placebo-controlled clinical trial. The study found no serious adverse events and a significant effect on unstimulated SFR four months post MSC(AT) therapy in the treated group [19]. This is also supported by a recent study by Lynggaard et al. [16]. However, the long-term effects of intraglandular MSC therapy in humans remain divergent [18, 42].

As a possible mode of action, Lynggaard et al. also investigated the regenerative effects of intraglandular allogeneic MSC(AT) therapy on the salivary proteome [43]. They observed an increase in proteins associated with tissue regeneration post transplantation, yet the salivary proteome did not return to a healthy state when compared to healthy controls [43].

This review is limited by the heterogeneity of methodologies and limited long-term data, hindering definite conclusions. The included studies varied in relation to included species, strains, origin of MSCs, delivering methods, radiation and follow-up regimen and study design. Prospects lie in optimizing challenges related to standardization of MSC therapy such as delivery methods, origin, and refining dosage protocols. Also, the radiotherapy regimens in the preclinical in vivo models were standardized and did not mirror those used in head and neck cancer patients. This lack of resemblance could potentially influence the effectiveness of MSC therapy in a clinical setting.

In conclusion, this systematic review and meta-analysis showed a significant effect of MSC therapy for restoring SG functioning and regenerating SG tissue following radiotherapy in preclinical in vivo studies. No serious adverse events were identified and intraglandular transplantation performed better effect than systemic transplantation. MSC therapy holds significant therapeutic potential in the treatment of radio-induced xerostomia and hypofunction, but comprehensive, randomized, clinical trials in humans are required to ascertain their efficacy in a clinical setting.

### Electronic Supplementary Material

Below is the link to the electronic supplementary material.

Supplementary Material 1

Supplementary Material 2

## Data Availability

Not applicable.
