# Supplementary material for: Mesenchymal Stromal/Stem Cell Therapy Improves Salivary Flow Rate in Radiation-Induced Salivary Gland Hypofunction in Preclinical in vivo Models: A Systematic Review and Meta-Analysis
Source: Stem Cell Rev Rep. 2024 Mar 2;20(4):1078–92. doi: 10.1007/s12015-024-10700-y (PMC11087340; doi:10.1007/s12015-024-10700-y)
Supplement: Supplementary file 1 — Supplementary Material 1 [file 12015_2024_10700_MOESM1_ESM.docx]

**Supplementary Results**

**Table 1.** Random-effects model sub analyses on categorical heterogeneity factors.

| **Subgroup analysis** | **Classifications** | **No. of studies** | **SMD** | **95% CI** | **p-value*** |
| --- | --- | --- | --- | --- | --- |
| **Species** | Mice | 10 | 6.3 | 1.8-10.8 | 0.63 |
|  | Rats | 2 | 13.4 | -132.0-158.7 |  |
|  | Miniature pigs | 1 | 4.7 | 1.7-7.6 |  |
| **Strain** | NOD.SCID-PrckSCID | 1 | 22.9 | 19.0-26.9 | <0.001* |
|  | C57BL/6 | 3 | 2.9 | 0.1-5.7 |  |
|  | Whistar | 1 | 25.4 | 15.2-35.6 |  |
|  | CH3 | 6 | 5.0 | 1.6-8.4 |  |
|  | Spraque-Dawlay | 1 | 2.5 | 1.8-3.2 |  |
|  | Missing | 1 | 4.7 | 1.8-7.6 |  |
| **Sex** | Female | 9 | 4.3 | 2.1-6.4 | 0.47 |
|  | Male | 2 | 13.4 | -132.0-158.7 |  |
|  | Unknown | 2 | 13.3 | -107.7-134.3 |  |
| **Administration route** | Intraglandular | 10 | 8.6 | 2.9-14.2 | 0.01* |
|  | Intravenously | 3 | 2.1 | 1.2-3.0 |  |

*significant value.

**Table 2.** Meta-regression sub analyses on continuous heterogeneity factors

| **Subgroup** | **Estimate** | **95% CI** | **p-value*** |
| --- | --- | --- | --- |
| **Age, weeks** | -0.3 | -0.9-0.3 | 0.3 |
| **Radiation dose, Gy** | -0.7 | -0.7-2.1 | 0.3 |
| **Frequency of treatment** | -0.6 | -1.7-0.5 | 0.2 |
| **Time between radiation and first treatment** | 0.0 | -0.2-0.1 | 0.7 |

*significant value.

**Table 3.** Human study characteristic.

| **Author (year)** | **Study design** | **Groups** | **Irradiation** | **Days from radiation to MSC treatment** | **MSC type, concentration and administration route** | **Statistical analysis** | **Functional outcome** | **Molecular outcome** |
| --- | --- | --- | --- | --- | --- | --- | --- | --- |
| Grønhøj, C. et al. (2018) | Prospective, randomized, blinded, controlled phase 1/2 trial | 1. placebo, n = 30  2. MSCs(AT), n = 30 | Mean Gy dose to single gland between 11.4 Gy and 71 Gy. 27 patients received both RT and chemotherapy, 3 received only RT. | Between 2.8 and 6.5 years. Median interval was 4.1 years. | MSC(AT)h (autologous).  Dose was 2.8 x 10^6^ cells x the volume of the gland (cm^3^).  Intraglandular injection, ultrasound guided (submandibular glands) | Sample size was calculated using a non-paired *t* test.  ﻿Within-group comparisons were performed with the Wilcoxon signed-rank test, and between-group comparisons were performed with the Mann-Whitney U test. Nonparametric statistics was evaluated by Shapiro-Wilks tests. Significance level was P < .05. | Unstimulated whole SFR was significantly increased in group 2 compared to baseline at both one-month and four months after treatment. Control group did not see this increase. The net scores between the two groups were similar. Group 2 showed significant improvements in patient reported outcome measures such as VAS score and xerostomia questionnaire regarding thirst and oral dryness. No adverse events in group 2 at 1 year. | 19 samples were evaluated, 11 were deemed not suitable for evaluation. A significant increase in serous gland tissue in group 2 was found compared to group 1. ﻿No significant differences was observed in the fractions of mucinous to serous tissue, mucinous tissue, glandular tissue adipose tissue. |

**Figure 2. Evaluation of reporting quality according to ARRIVE guidelines**. *: 1=Study design; 2= Sample size; 3= Inclusion and exclusion criteria; 4= Randomization, **none of the studies reported randomization method; 5=Blinding; 6=Outcome measures; 7=Statistical methods; 8=Experimental animals; 9=Experimental procedures; 10=Results. One point was given if the criteria was sufficiently fulfilled, maximum total quality score possible was 10.

| **Author (Year)** | **1*** | **2*** | **3*** | **4*** | **5**** | **6*** | **7*** | **8*** | **9*** | **10*** | **Total quality score** |
| --- | --- | --- | --- | --- | --- | --- | --- | --- | --- | --- | --- |
| Lin, C et al. (2011) | 1 | 1 | 0 | 1** | 0 | 1 | 0 | 1 | 1 | 1 | 7 |
| Kojima, T. et al. (2011) | 1 | 1 | 0 | 1** | 0 | 1 | 1 | 1 | 1 | 1 | 8 |
| Jeong, J. et al. (2013) | 0 | 0 | 0 | 1** | 0 | 1 | 1 | 1 | 0 | 1 | 5 |
| Lim, J. et al. (2013, oral) | 1 | 1 | 0 | 1** | 1 | 1 | 1 | 1 | 1 | 1 | 9 |
| Lim, J. et al. (2013, PLoS ONE) | 1 | 1 | 0 | 1** | 1 | 1 | 1 | 1 | 1 | 1 | 9 |
| Xiong, X. et al. (2014) | 1 | 1 | 0 | 1** | 0 | 1 | 1 | 1 | 1 | 1 | 8 |
| Chen, Y. and Niu, Z. et al. (2014) | 1 | 1 | 0 | 1** | 0 | 1 | 1 | 0 | 1 | 1 | 7 |
| An, H. et al. (2015) | 1 | 1 | 0 | 1** | 1 | 1 | 1 | 1 | 1 | 1 | 8 |
| Li, Z. et al. (2015) | 1 | 1 | 0 | 1** | 0 | 1 | 1 | 1 | 1 | 1 | 8 |
| Wang, Z. et al. (2016) | 1 | 1 | 0 | 1** | 1 | 1 | 1 | 1 | 1 | 1 | 9 |
| Wang, Z. et al. (2017) | 1 | 1 | 0 | 1** | 1 | 1 | 1 | 1 | 1 | 1 | 9 |
| Choi, J. et al. (2018) | 1 | 1 | 0 | 1** | 1 | 1 | 1 | 1 | 1 | 1 | 9 |
| Shin, H. et al. (2018) | 1 | 1 | 0 | 1** | 0 | 1 | 1 | 1 | 1 | 1 | 8 |
| Shin, H. et al. (2018) | 1 | 1 | 0 | 1** | 0 | 1 | 1 | 1 | 1 | 1 | 8 |
| Elsaadany, B. et al. (2019) | 1 | 1 | 0 | 1** | 1 | 1 | 1 | 1 | 1 | 1 | 9 |
| Mulyani, S. et al. (2019) | 1 | 1 | 0 | 1** | 0 | 1 | 1 | 1 | 0 | 1 | 7 |
