# Supplementary material for: Mesenchymal Stromal/Stem Cell Therapy Improves Salivary Flow Rate in Radiation-Induced Salivary Gland Hypofunction in Preclinical in vivo Models: A Systematic Review and Meta-Analysis
Source: Stem Cell Rev Rep. 2024 Mar 2;20(4):1078–92. doi: 10.1007/s12015-024-10700-y (PMC11087340; doi:10.1007/s12015-024-10700-y)
Supplement: Supplementary file 2 — Supplementary Material 2 [file 12015_2024_10700_MOESM2_ESM.docx]

**Stem Cell Reviews and Reports**

**Professor Mariusz Z. Ratajczak, Editor-in-Chief**

Dear Editor,

I am pleased to resubmit this report entitled: *”Mesenchymal stromal/stem cell therapy improves salivary flow rate in radiation-induced salivary gland hypofunction in preclinical in vivo models: a systematic review and meta-analysis.”* We hope you will consider our manuscript for publication in Stem Cell Reviews and Reports. We have thoroughly revised the manuscript according to the changes suggested by the reviewer, as reported in our *Response to reviewer*. We have:

- Implemented a substantial reduction in emphasizing human studies, apparent in both the results, the discussion, and Table 1
- Revised the “n=” throughout the manuscript and specifically stated if the number refers to number of studies or number of experimental animals
- Added the suggested references on human studies
- Added quality assurance of radiation delivery in data extraction and Table 1.
- Added a detailed description of how SMD is calculated
- Added details on blinding of the outcome assessment
- Specified records excluded in Figure 1.

The objective of this investigation was to evaluate the safety and efficacy of mesenchymal stem cell therapy in restoring salivary gland function post radiation-induced damage through preclinical in vivo studies. This research holds significant value in refining clinical trials and understanding the potential implications for treating radiation-induced salivary gland hypofunction.

We anticipate that our findings will be of considerable interest to Stem Cell Reviews and Reports readers offering relevant and new insights into the clinical viability and mechanisms underlying mesenchymal stem cell therapy for radiotherapy-induced xerostomia. We certify that the research presented in the paper is original and not presently under consideration for publication elsewhere. All the authors have approved the complete content of the paper prior to submission to Stem Cell Reviews and Reports. The authors declare no conflicts of interest.

On behalf of the authors,

Sincerely,

*Amanda-Louise Fenger Carlander
Department of Otorhinolaryngology, Head and Neck Surgery and Audiology,
University Hospital Copenhagen, Denmark
Inge Lehmanns Vej 8, 2100 Copenhagen
E-mail: Amanda-louise.fenger.carlander@regionh.dk
Phone: +45 30 22 31 02*
